# Cerebral Blood Flow Deviations in Critically Ill Patients: Potential Insult Contributing to Ischemic and Hyperemic Injury

**DOI:** 10.3389/fmed.2020.615318

**Published:** 2021-01-20

**Authors:** Marat Slessarev, Ossama Mahmoud, Christopher W. McIntyre, Christopher G. Ellis

**Affiliations:** ^1^Department of Medicine, Western University, London, ON, Canada; ^2^Department of Medical Biophysics, Western University, London, ON, Canada; ^3^Brain & Mind Institute, Western University, London, ON, Canada; ^4^Department of Computer Science, Western University, London, ON, Canada; ^5^Robarts Research Institute, Western University, London, ON, Canada

**Keywords:** critical illness, cerebral blood flow, autoregulation, carbon dioxide, blood pressure

## Abstract

**Background:** Ischemic and hyperemic injury have emerged as biologic mechanisms that contribute to cognitive impairment in critically ill patients. Spontaneous deviations in cerebral blood flow (CBF) beyond ischemic and hyperemic thresholds may represent an insult that contributes to this brain injury, especially if they accumulate over time and coincide with impaired autoregulation.

**Methods:** We used transcranial Doppler to measure the proportion of time that CBF velocity (CBFv) deviated beyond previously reported ischemic and hyperemic thresholds in a cohort of critically ill patients with respiratory failure and/or shock within 48 h of ICU admission. We also assessed whether these CBFv deviations were more common during periods of impaired dynamic autoregulation, and whether they are explained by concurrent variations in mean arterial pressure (MAP) and end-tidal PCO_2_ (PetCO_2_).

**Results:** We enrolled 12 consecutive patients (three females) who were monitored for a mean duration of 462.6 ± 39.8 min. Across patients, CBFv deviated by more than 20–30% from its baseline for 17–24% of the analysis time. These CBFv deviations occurred equally during periods of preserved and impaired autoregulation, while concurrent variations in MAP and PetCO_2_ explained only 13–21% of these CBFv deviations.

**Conclusion:** CBFv deviations beyond ischemic and hyperemic thresholds are common in critically ill patients with respiratory failure or shock. These deviations occur irrespective of the state of dynamic autoregulation and are not explained by changes in MAP and CO_2_. Future studies should explore mechanisms responsible for these CBFv deviations and establish whether their cumulative burden predicts poor neurocognitive outcomes.

## Introduction

Critically ill patients have a high prevalence of acute (delirium) (1) and long-term cognitive impairment (2, 3). Ischemic and hyperemic injury have both emerged as plausible biologic mechanisms responsible for these disturbances due to common finding of ischemic lesions on neuroimaging (4, 5) and histopathology (6, 7), disturbed autoregulation (8–11) and circulatory stress (12, 13) in this patient population. Spontaneous deviations in cerebral blood flow (CBF) beyond ischemic and hyperemic thresholds earlier in the course of critical illness may represent an insult that contributes to this brain injury, especially if it accumulates over time and occurs during periods of impaired autoregulation.

While spontaneous variation in cerebral blood flow (CBF) are common in both healthy adults (14, 15) and patients with primary brain pathology (16, 17), the proportion of time that CBF deviated beyond ischemic and hyperemic thresholds in critically ill patients without brain injury has not been well-described. Furthermore, the association of these spontaneous CBF deviations with periods of impaired autoregulation and spontaneous variations in mean arterial pressure (MAP) and CO_2_ have not been described.

In this study, we aimed to determine the proportion of time that CBF deviated beyond previously reported ischemic and hyperemic thresholds in a cohort of critically ill patients without brain injury early in the course of their critical illness. In secondary analysis, we assessed whether these deviations in CBF were more common during periods of impaired dynamic autoregulation. Finally, we used regression analysis to determine the relative contribution of variations in MAP and end-tidal partial pressure of CO_2_ (PetCO_2_) to these deviations in CBF. We hypothesized that: (1) CBFv deviations are common, (2) CBFv deviation are more likely occur during periods of impaired dynamic autoregulation, and (3) >80% of variability in CBFv can be explained by concurrent variations in MAP and PetCO_2_.

## Materials and Methods

### Study Design and Participants

This was a single-center prospective observational cohort study that was approved by Western University Health Sciences Research Ethics Board (protocol number 106955) and carried out at the Critical Care Trauma Center, London Health Sciences Center. We enrolled consecutive adult (age ≥ 18 years) patients within 48 h of admission to the ICU who presented with a diagnosis of respiratory failure requiring mechanical ventilation > 24 h and/or shock (defined as the requirement for vasopressors). All participants had an indwelling arterial catheter for continuous monitoring of arterial blood pressure. We excluded patients presenting with primary neurologic diagnosis or known cerebrovascular diseases. Signed informed consent was obtained from patient's substitute decision maker prior to enrollment in the study. The measurements were made on the day of enrollment into the study.

### Cerebral Blood Flow Monitoring

We used transcranial Doppler to measures middle cerebral artery blood flow velocity (CBFv) as an indicator of global CBF. We used 2-MHz transcranial Doppler probes (ST3, Spencer Technologies, USA) to insonate one/both middle cerebral arteries through the transtemporal window. The middle cerebral arteries were identified using standard technique described in the literature (18, 19), and the probes were fixed in place using provided head frame (Spencer Technologies, Redmond, WA, USA). Adequacy of CBFv signals was continuously monitored throughout the 8 h of observation by study investigators, and probes were readjusted as needed to ensure same angle of insonation within the same patient and good signal power. When CBFv recording was available from both right and left middle cerebral artery, we used the average of the two signals.

Blood flow velocity was sampled and recorded continuously at 125 Hz on ST3 device.

### Hemodynamic and CO_2_ Monitoring

Arterial blood pressure was monitored using an indwelling arterial catheter placed into radial, brachial or femoral arteries. CO_2_ was monitored using continuous in-line capnography module integrated into the ventilator circuit (S/5, Datex-Ohmeda, GE Healthcare, USA). The transduced arterial pressure and expired CO_2_ were displayed on clinical monitors (S/5, Datex-Ohmeda, GE Healthcare, USA) and continuous waveforms were recorded at 300 Hz on a laptop computer using S5-collect software (Datex-Ohmeda, GE Healthcare, USA).

### Study Protocol and Data Collection

Following enrollment in the study, baseline demographic, comorbidity and clinical data were obtained from patients' medical charts and recorded in dedicated case report forms. Waveform CBFv, arterial pressure and CO_2_ data were recorded continuously for up to 8 h and exported as coma-separated values for offline analysis. The data was then uploaded into a custom waveform viewer (LabView, National Instruments, Austin, Texas, USA), where it was aligned using time stamps and processed to ensure adequacy of all signals. Artifacts and sections with poor signals (e.g., CO_2_ and blood pressure calibration artifacts, coughing, movement resulting in poor signals) were excluded from the analysis. The software then used LabView peak detector function to identify end-tidal CO_2_ (PetCO_2_) and systolic, diastolic and mean arterial pressures (MAP) and CBFv, which were then checked by investigators to ensure appropriate identification of these values from continuous traces. Inappropriately picked values were manually readjusted or excluded from the analysis (e.g., double end-tidal picking due to ventilator asynchrony). Resulting data were averaged over 5 s intervals and exported as comma-separated values for further analysis in Python and Excel. To allow comparison between participants, CBFv was expressed as percent change from participant's baseline. To calculate primary outcome, we computed the proportion of total recording time that CBFv deviated from baseline above or below thresholds of 5, 10, 15, 20, 25 and 30% (see Supplementary Figure SM-1). Given that CBF ischemia thresholds have not yet been defined in this patient population, we used a range of thresholds that encompasses previously reported thresholds for ischemia in other patient populations (20), as well as thresholds that would equate to white matter ischemia (21, 22).

### Dynamic Autoregulation Analysis

For our secondary analysis, we computed clinically validated index of dynamic autoregulation (Mxa) (23) by calculating Spearman's correlation coefficient between CBFv and MAP values within a moving time window advanced in 1-min steps. In keeping with previous definitions, we defined disturbed autoregulation as Mxa values >0.3 that were statistically significant (*p* < 0.05), and intact autoregulation as Mxa values <0.3 (including negative values) (24). Spearman's method was used instead of Pearson's as we could not assume the linearity in the relationship between MAP and CBFv (25). To determine the impact of window length on calculation of Mxa, we varied the window length across a range of values (5, 10, 15, 20, 30, and 60 min). Mxa was only computed in windows that had at least 5 mmHg variation in MAP (26). We expressed the duration of disturbed autoregulation for each patient as a fraction of the total analysis time minus the time that Mxa could not be assessed (see Supplementary Figure SM-2 for sample calculation of Mxa from MAP and CBFv data).

### Relationship Between CBFv Deviations and State of Dynamic Autoregulation

To establish whether the duration of CBFv deviations beyond thresholds were longer in duration if dynamic autoregulation was impaired, we compared the fraction of time that CBFv deviated above and below thresholds when autoregulation was impaired vs. when it was preserved. In each patient, we used 5-min moving windows to compute Mxa as described above in order to assess whether autoregulation was preserved or impaired. We then computed the time (as a fraction of the 5-min window) that CBFv deviated above/below CBF thresholds within the same window. We summarized the data within patients by comparing the cumulative time that CBFv deviated above/below CBF thresholds depending on the state of dynamic autoregulation (preserved vs. impaired). We then compared results across patients. We used two-way ANOVA and Sidak's multiple comparison test to assess statistical difference between groups.

### Regression Analysis to Determine the Relative Impact of MAP and CO_2_ on CBFv

To establish the contribution of variations in MAP and CO_2_ to observed deviations in CBFv, we performed a multiple linear regression analysis with MAP and CO_2_ as independent variables and CBFv as a dependent variable (Supplementary Figure SM-3). Regression analysis was done within the same moving time window as Mxa that was advanced at 1-min intervals. To determine the impact of window length on the outcome of regression analysis, we varied it across a range of values (5, 10, 15, 20, 30, and 60 min). Regression analysis was completed only in windows that had at least 5 mmHg variation in MAP and at least 1 mmHg variation in CO_2_ (the lower magnitude of variation in CO_2_ was accepted since CBFv is exquisitely sensitive to changes in CO_2_, ~3–5% change per mmHg change in CO_2_) (27). Regression coefficients of determination (R^2^) for each window length were summarized in each patient and across patients.

### Statistical Analysis

Statistical analysis was performed using the GraphPad Prism software version 8.3 for macOS (GraphPad Software, San Diego, CA, U.S.A.). Categorical variables are reported as counts and percentages of total. Continuous data were analyzed for normal distribution using the Shapiro-Wilk normality test and were reported as mean and standard deviation for normally distributed data; otherwise, data were summarized using median and interquartile range. We used 2-way ANOVA and Sidak's multiple comparison test to assess for difference in the duration of CBFv deviations above/below thresholds between windows with preserved vs. impaired autoregulation. For all statistical comparisons, a *p*-value of <0.05 was considered significant.

## Results

### Patients Baseline Characteristics

**Figure 1:**
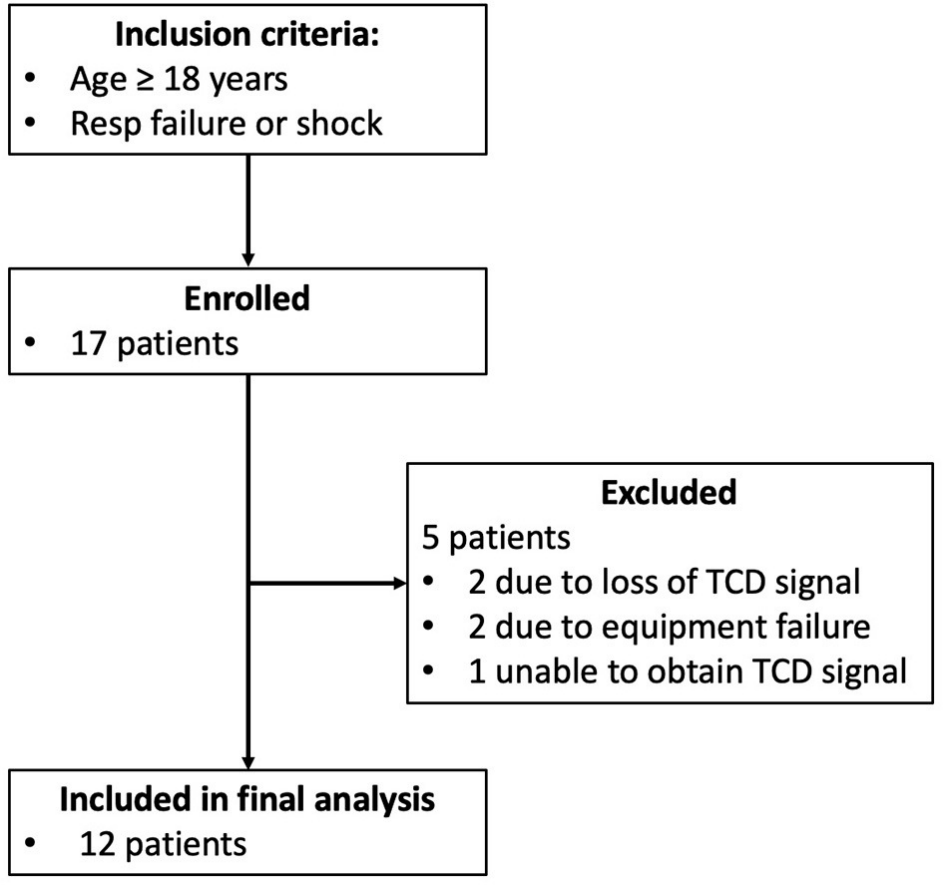
Patient enrollment flow diagram.

**Figure 2:**
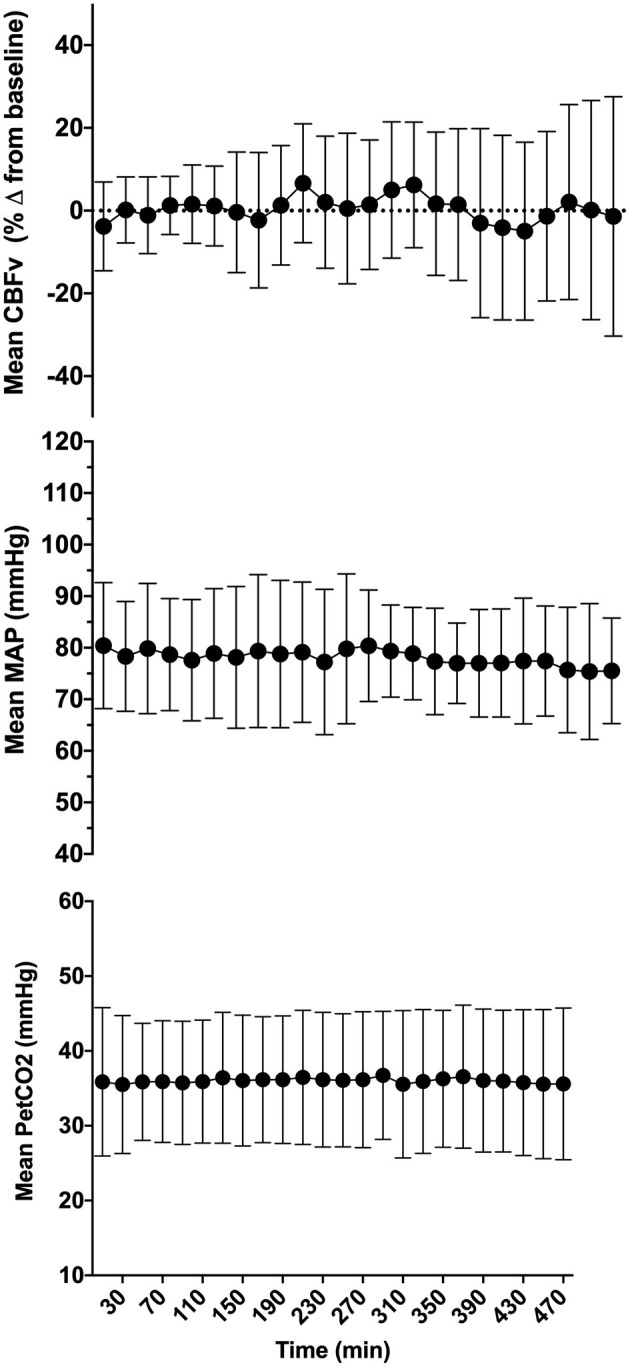
Mean cerebral blood flow velocity (CBFv), mean arterial pressure (MAP), and end-tidal PCO_2_ (PetCO_2_) across all patients included in the final analysis. Error bars represent standard deviation. CBFv was expressed as percent change from baseline to enable comparison across patients.

Seventeen patients met the inclusion criteria and were enrolled in the study. Five patients were excluded (two due to loss of signals, two due to recording equipment malfunction, one due to inability to obtain transcranial Doppler signal), leaving 12 patients in the final analysis (Figure 1). Patients baseline characteristics are summarized in Table 1. Median (interquartile range, IQR) age was 62 (43, 80) years, there were fewer females in our cohort (3/12 patients), and all patient had respiratory failure and required vasopressors for shock. The most common comorbidities included dyslipidemia, obstructive lung disease, and cardiovascular disease. Median (IQR) SOFA score was 10 (7, 12), MODS score was 6 (5, 8) and arterial to end-tidal PCO_2_ difference was 8 (4, 17) mmHg. Across patient, the mean duration of the observation period was 462.6 ± 39.8 min. After removal of artifacts, 379.2 ± 72.6 min was available for analysis across patients (Figure 2). During this period, the mean CBFv was 0.4 ± 17.1% from baseline, MAP 78.1 ± 11.5 mmHg, and PetCO_2_ was 36.0 ± 8.7 mmHg.

**Table 1:** Patient baseline characteristics.

Number of patients	12
Age (years)	62 (43, 80)
Females, *n* (%)	3 (25)
MODS score	6 (5, 8)
NEMS score	39 (28, 39)
SOFA score	10 (7, 12)
Arterial-end-tidal PCO_2_ difference (mmHg)	8 (4, 17)
**Admission diagnosis**	
Respiratory failure requiring mechanical ventilation	12 (100)
Shock requiring vasopressors	12 (100)
**Ventilation mode**	
Spontaneous	4 (33)
Controlled	8 67)
**Comorbidities**	
Hypertension	3 (25)
CAD	3 (25)
CHF	3 (25)
Afib	2 (17)
DM	3 (25)
Dyslipidemia	4 (33)
Obstructive lung disease	4 (33)
Restrictive lung disease	1 (8)

*Values are expressed as counts (%) or as medians (IQR). MODS, multiple organ dysfunction syndrome; NEMS, nine equivalents of nursing manpower; SOFA, sequential organ failure assessment; CAD, coronary artery disease; CHF, congestive heart failure; Afib, atrial fibrillation; DM, diabetes mellitus*.

### Duration of CBFv Deviations Above and Below Thresholds

**Figure 3:**
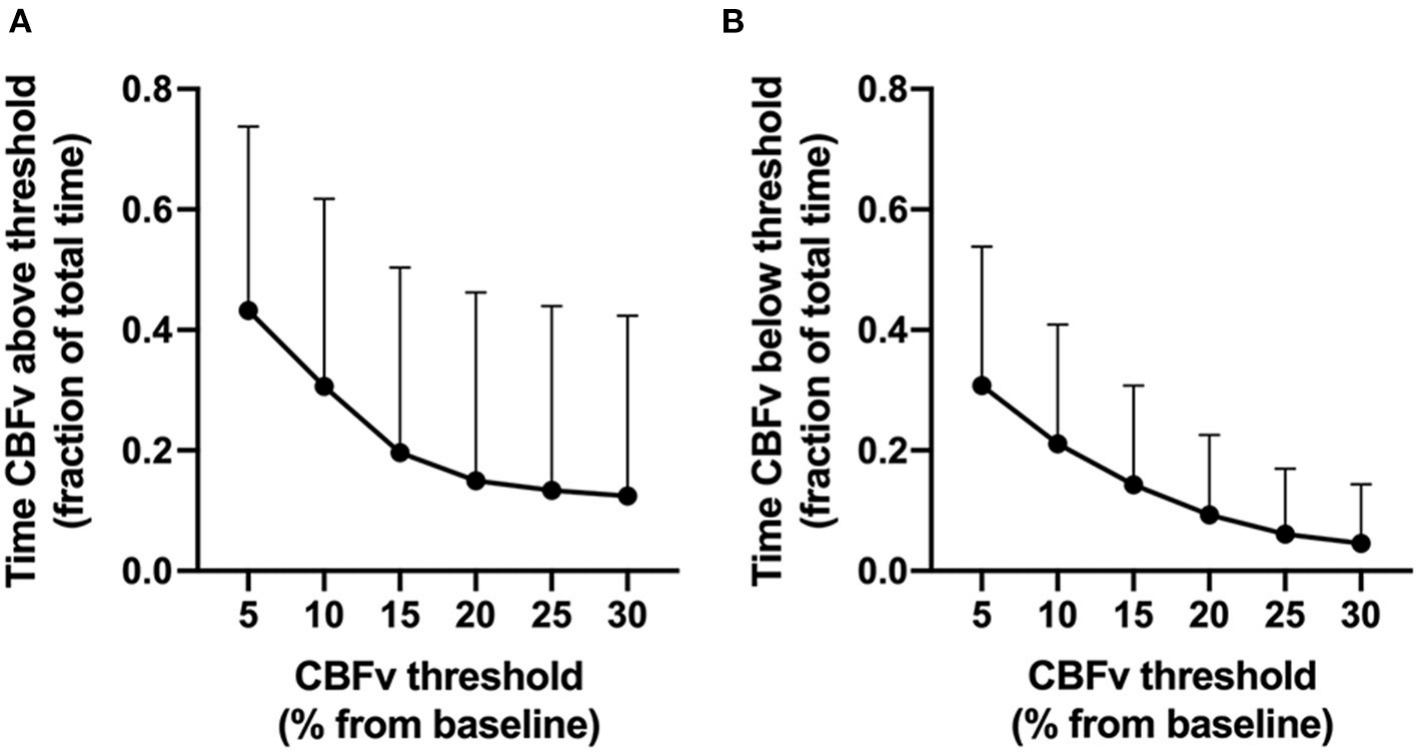
Proportion of analysis time that cerebral blood flow velocity (CBFv) deviated from baseline (y axis) above **(A)** or below **(B)** CBFv thresholds (x-axis). Error bars represent standard deviations.

CBFv deviated by more than 10, 20, and 30% from baseline for approximately 52, 24, and 17% of the total analysis time across patients (Supplementary Table SM-2). CBFv deviations from baseline above thresholds were more common that CBFv deviations below thresholds (Figure 3). CBFv deviated by 10–20% below baseline for an average of 9–21% of the total analysis time.

### Dynamic Autoregulation

**Figure 4:**
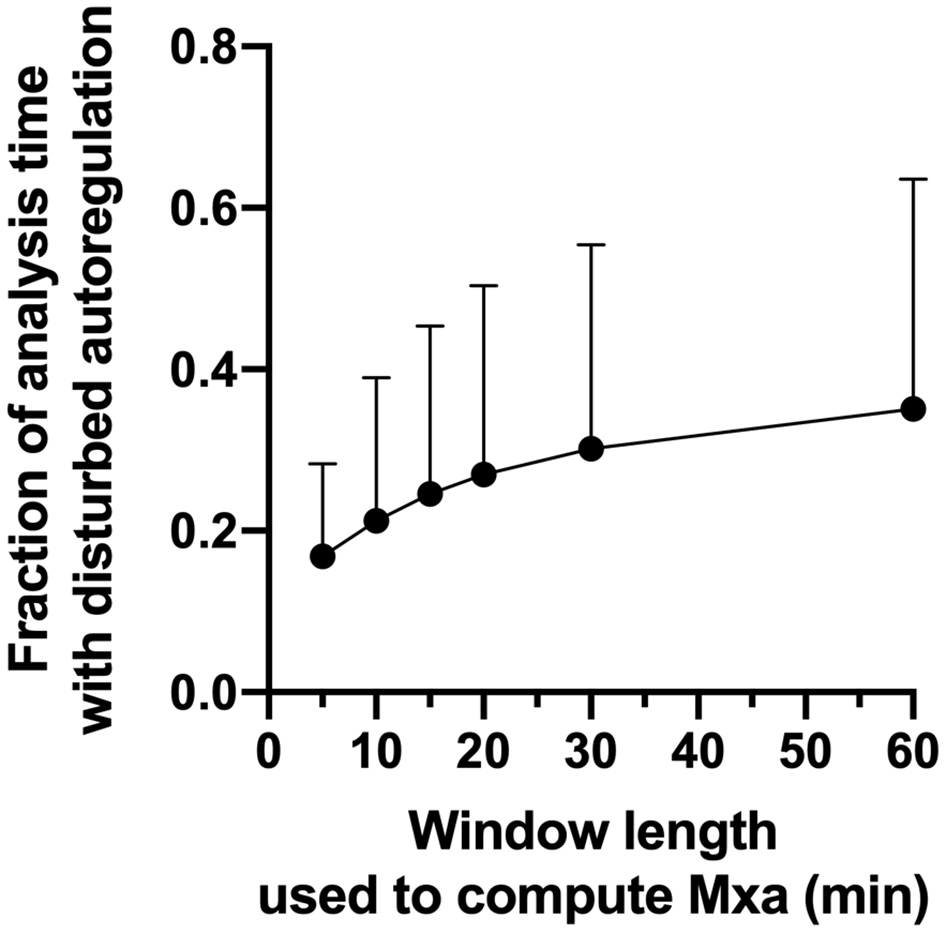
Fraction of the analysis time with disturbed autoregulation as a function of window length used to calculate Mxa. Circles are mean values, Error bars are SD.

Across patients, dynamic autoregulation was disturbed 20–35% of the observation time depending on the length of the window used to calculate Mxa (Supplementary Table SM-3). In keeping with the previous studies (25), longer window length was associated with the larger fraction of observation time with disturbed autoregulation (Figure 4).

### Relationship Between Duration of CBFv Deviations and State of Dynamic Autoregulation

**Figure 5:**
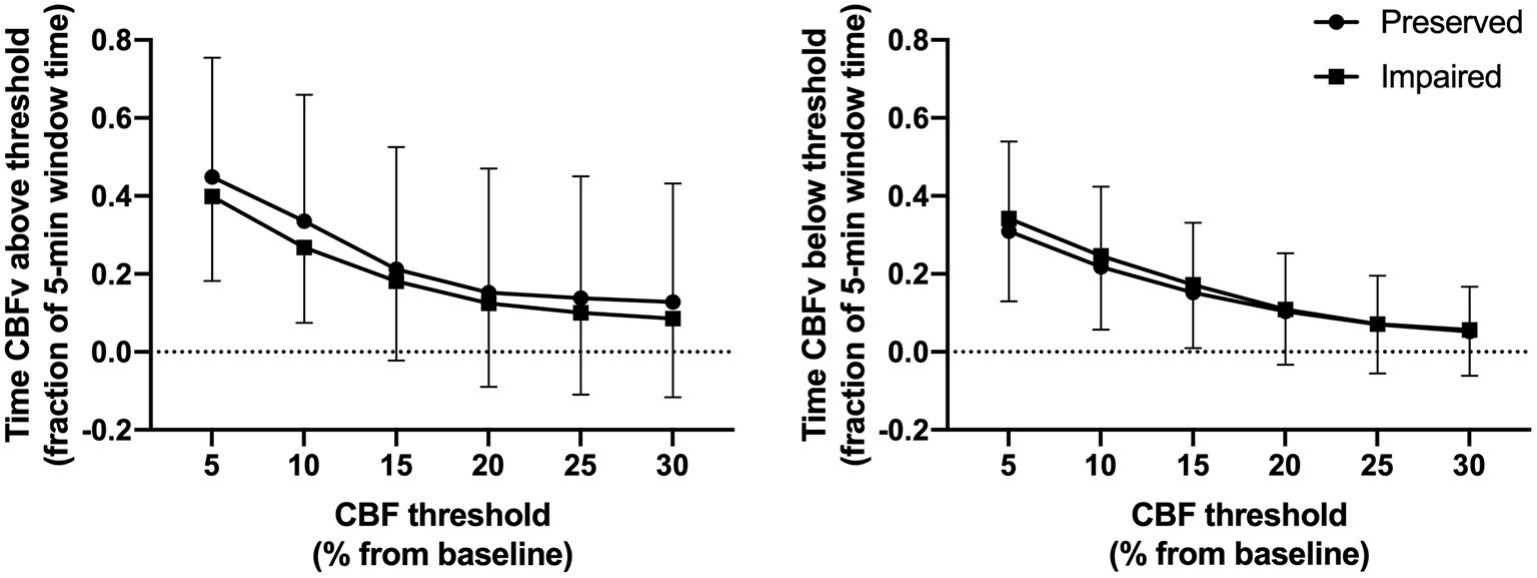
Relationship between CBFv deviations above/below thresholds and state of dynamic autoregulation across patients. Circles denote fraction of the 5-min window used to compute Mxa when autoregulation was preserved. Squares denote fraction of the 5-min window used to compute Mxa when autoregulation was impaired.

Across patients, the state of dynamic autoregulation had no impact on the duration of CBFv deviations above or below thresholds (Figure 5, and Supplementary Table SM-4). While the proportion of time that CBFv deviated above/below CBF thresholds varied across patients (Supplementary Figures SM-4, SM-5), the state of autoregulation within patients had little effect on the duration of these CBFv deviations. In fact, in some patients (e.g., patient 7) CBFv deviations were more common during periods of preserved autoregulation. In another patient (patient 1), CBFv deviations above thresholds were longer during periods of impaired autoregulation, while those below thresholds were longer during periods of preserved autoregulation. While CBFv deviations during periods of impaired autoregulation are expected, the surprising finding in this analysis was that CBFv deviations occurred for substantial proportion of time during periods of preserved dynamic autoregulation. For example, during periods of preserved autoregulation, across patients CBFv deviated by 10–20% above baseline for 15–34% of observed time and by 10–20% below baseline for 10–22% of observed time.

### Regression Analysis

**Figure 6:**
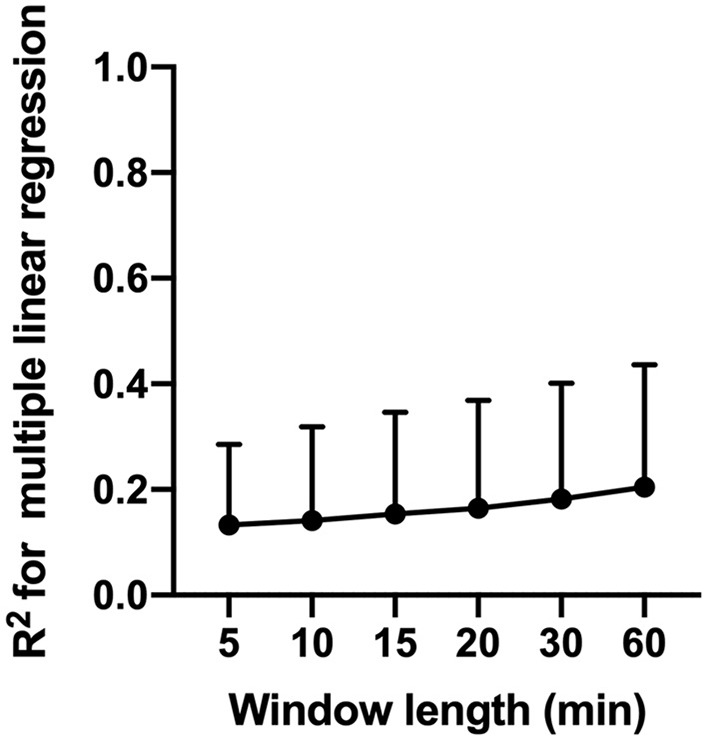
Results of multiple linear regression analysis with MAP and CO2 and independent variables and CBFv as a dependent variable. The figure shows changes in coefficient of determination (R2) as a function of window length use for regression analysis.

Multiple linear regression analysis with MAP and CO_2_ as independent variables and CBFv as a dependent variable yielded very low R^2^ values across patients. Across patients, The R^2^ varied from 0.13 ± 0.15 to 0.21 ± 0.23 depending on the window length used to complete regression analysis (Figure 6). Results of the regression analysis within individual patients is summarized in Supplementary Table SM-1.

## Discussion

### Main Findings

Our study shows that in critically ill patients with respiratory failure or shock, CBFv deviates by more than 20–30% from its baseline for 17–24% of the analysis time. There were more CBFv deviations above than below the baseline (15 ± 31 vs. 9 ± 13% of the observation time). Contrary to our hypotheses, these CBFv deviations were not more common during periods of impaired dynamic autoregulation (which was impaired 20–35% of the observation time in our cohort), nor were they fully explained by associated variations in MAP and CO_2_. Given that CBF thresholds used in our study are similar to those associated with ischemic injury and neurologic symptoms from previous studies in cardiac surgery patients, our findings represent biologically plausible insult that may lead to CBF-mediated brain injury in critically ill patients independently from the state of dynamic autoregulation or changes in MAP and CO_2_.

### CBF Deviations in Healthy Subjects and Patients With Neurologic Disorders

To our knowledge this is the first study to quantify the magnitude and duration of spontaneous CBFv deviations beyond specific thresholds in patients with respiratory failure or shock. Spontaneous deviations in CBF have been previously described in healthy participants and in patients with neurologic disorders and range between 11 and 14% from baseline (16). In patients with traumatic brain injury, Davie et al. used cerebral oximetry to show that 12 of 18 patients experience an episode of cerebral desaturation of 7–10% from baseline, which is comparable to the magnitude seen in our study.

### What CBF Thresholds Are Associated With Brain Injury?

While much work has been done on ischemic thresholds in animal models and human studies of stroke, information in other clinical populations is less clear. Global CBF reduction by 20–30% from baseline has been associated with impairment of brain function including loss of EEG signal in humans (28), failure of cellular protein synthesis (29), and cognitive dysfunction (30). In cardiac surgery patients, reduction of cerebral oxygenation by 7–10% from baseline is associated with major clinical complications including stroke and delirium (31) and development of new ischemic lesions on neuroimaging (20). Interestingly, in the latter study, the majority (70%) of new lesions developed in the watershed areas of the brain (20), suggesting that sub-cortical white matter tracts are at higher risk of this subclinical ischemic injury. Since critical illness is associated with the disruption of the brain blood barrier (32), hyperemic response may also contribute to injury by increasing intracranial pressure (33) and contributing to formation of brain edema, especially in settings of disturbed autoregulation (34).

### The Duration of CBF Deviations Beyond Thresholds – “Time Is Brain”

In stroke literature, the concept of “time is brain” is well established. While ischemic injury in stroke may be an extreme case of neuronal injury, similar evidence is evolving for more subtle injury in cardiac surgery patients. Duration of time that regional cerebral oxygenation deviates by ~7–10% below baseline is associated with greater number of major post-operative clinical complications (including stroke, delirium, respiratory and renal failure), longer duration of mechanical ventilation, ICU and hospital length of stay (31), and development of new ischemic lesions primarily in watershed areas of the brain (20). In the latter study, the median duration that cerebral oxygenation was 10% below baseline was ~5% of the total observation time, highlighting that even short cumulative duration of modest impairment in cerebral perfusion can result in subclinical watershed ischemic brain injury. This duration of cerebral perfusion deviation into ischemic zone is comparable to our study, where CBFv deviated by more than 20–30% below baseline for a mean 5–9% of the observation time. The importance of time in development of brain dysfunction is further highlighted by a recent study from critically ill patients showing that the cumulative duration of dysfunctional cerebral autoregulation is associated with the development of ICU delirium (25).

### Cerebral Autoregulation and Deviations in CBFv

In our study, dynamic cerebral autoregulation was disturbed 20–35% of the observation time, depending on the length of the window used to compute Mxa (Figure 4). However, the disturbance in autoregulation had no impact on the duration of CBFv deviations above/below thresholds (Figure 5). In fact, during periods of preserved autoregulation CBFv deviated by 10–20% above/below baseline for 10–34% of observation time. These findings suggest that preserved dynamic autoregulation does not prevent substantial variations in CBFv. This is surprising, given that prior studies have demonstrated association of disturbed dynamic autoregulation with poor clinical outcomes in critically ill patients with head injury, sepsis and delirium (8, 24, 25, 35, 36). However, association does not imply causation. In fact, impaired autoregulation by itself does not represent an ischemic or hyperemic insult. Ischemia and hyperemia require variation in CBFv of magnitude and duration similar to what we showed in this study. Since we demonstrated that CBFv deviations occur irrespective of autoregulation state, it is possible that prior studies demonstrating association between impaired autoregulation and poor clinical outcomes are due to association between CBFv and Mxa, given that CBFv is a co-variate used in computation of Mxa. Furthermore, since changes in MAP and CO_2_ only explained ~20% of variability in CBFv in our study, it is possible that variables and mechanisms other than MAP, CO_2_ and autoregulation are involved in ischemic/hyperemic injury in critically ill patients. These may include changes in cardiac output (37) and regional cerebrovascular reactivity (38). In summary, our findings suggest that in critically ill patients prolonged deviations in CBFv from baseline can occur independently of disturbances in dynamic autoregulation. Whether cumulative burden of these CBFv deviations is an independent predictor of poor clinical outcomes requires further dedicated studies.

### Association of CBFv Deviations With MAP and CO_2_

Arterial blood pressure and CO_2_ are important determinants of CBF. Changes in MAP result in changes in cerebral perfusion pressure and CBF, especially if cerebral autoregulation is impaired. CBF is also exquisitely sensitive to arterial CO_2_, which can induce changes in CBF by altering cerebrovascular resistance (1 mmHg change in arterial PCO_2_ is associated with approximately 2–5% change in CBF in subjects with preserved cerebrovascular reactivity) (27). Given that both MAP and CO_2_ are important determinants of CBF, and since both of these variables fluctuate spontaneously in critically ill patients, we used multiple regression analysis to determine whether CBF deviations can be explained by concurrent changes in MAP and CO_2_.

Our analysis revealed that across patients, variation in MAP and CO_2_ explain only 13–21% of the observed variance in CBFv (Figure 6). These findings suggest that variables other than MAP and CO_2_ are responsible for a large proportion of the observed CBFv deviation. Alternative mechanisms responsible for observed CBFv deviations may include changes in cardiac output, which has been shown to be an important determinant of CBF independent of cerebral autoregulation and MAP (37, 39, 40), as well as changes in regional cerebral metabolism and cerebrovascular resistance. Our results highlight the complexity of CBF regulation in the critically ill patients and warrant further multimodal studies to delineate the relative contribution of various control mechanisms to observed deviations in CBF.

### Future Work

Future studies should assess whether the cumulative burden of observed deviations in CBFv from baseline is associated with clinically relevant outcomes including delirium and long-term cognitive impairment. Furthermore, given that after accounting for variations in MAP and CO_2_, ~80% of variance in CBFv remained unexplained, future work should concurrently assess additional variables that may affect CBF including cardiac output and measures of cerebral metabolism and regional cerebral perfusion. While tomographic imaging modalities such and PET and MRI would be ideal for these measurements, it may not be feasible or safe to transport critically ill patients outside of ICU for prolonged periods of monitoring using these tomographic modalities. Furthermore, given that assessment of dynamic autoregulation requires high frequency monitoring of CBF and MAP, the lower temporal resolution of MRI and PET may not be ideal for the assessment of dynamic autoregulation in these patients. Multimodal monitoring with transcranial Doppler, near infrared spectroscopy, diffuse correlation spectroscopy and non-invasive continuous cardiac output monitors is likely an optimal compromise to enable simultaneous assessment of global and regional CBF, regional cerebral metabolism, dynamic autoregulation and cardiac output at the bedside of critically ill patients. Our plans are to explore this approach in future studies.

### Limitations

Our study had several limitations. Due to the pilot nature of our study, we had a relatively small sample size and as a result of consecutive patient enrollment strategy our sample was not equally balanced between females and males. Despite these limitations, we were able to record CBFv, MAP and PetCO_2_ continuously in each patient for an average of 462.6 ± 39.8 min, which results in an average of 379.2 ± 72.6 min of data in each patient available for analysis after removal of artifacts. These long recordings enabled detailed examination of CBFv deviations and their relationship to dynamic autoregulation and variations in MAP and PetCO_2_ in individual patients. As a result, we were able to demonstrate that, similar to patients with traumatic brain injury (41), critically ill patients experienced 20–30% deviations in CBF from baseline for substantial periods of time. We also showed that our patients experienced substantial periods of disturbed dynamic cerebral autoregulation. To define impaired autoregulation, we used Mxa threshold of >0.3 based on prior literature from critically ill patients with neurologic illness, as well as those with sepsis, septic shock and sepsis-associate delirium (8, 24, 25, 35, 36). Future studies should establish Mxa thresholds that correspond to poor clinical outcomes in patients with respiratory failure and/or shock. We did not measure many variables than may impact CBFv including cardiac output (37) and temperature (42, 43). Future studies should incorporate these measurements as potential co-variates that may affect CBFv.

Another limitation of our study is that the use of transcranial Doppler limited our assessment of cerebral blood flow velocity to middle cerebral artery, and therefore represent only portion of global cerebral blood flow. However, the portable nature of transcranial Doppler relative to comprehensive tomographic modalities allowed its application at the bedside early in the course of critical illness when these patients are usually very unstable for transfer to neuroimaging department. Furthermore, its high temporal resolution made it an ideal tool for monitoring and discerning high frequency changes in CBFv over prolonged period of observation used in this study. Finally, whether the observed deviations in CBFv are associated with ischemic brain injury and brain dysfunction, or are simply an epiphenomenon, remains unknown. However, our study provides biologic rationale for future studies to establish whether observed deviations in CBF from baseline in this patient population are associated with imaging markers of brain injury or functional brain impairment.

## Conclusion

In critically ill patients with respiratory failure or shock, CBFv deviates by 20–30% from baseline for 17–24% of observation time early in the course of ICU admission. These deviations occurred both during periods of preserved and impaired autoregulation and were not fully explained by corresponding changes in MAP and CO_2_. Future studies should use multimodal neuromonitoring to establish the mechanisms responsible for these CBF deviations and assess whether cumulative burden of these deviations is associated with imaging markers of brain injury and poor neurocognitive outcomes.

## Data Availability Statement

The original contributions generated for this study are included in the article/Supplementary Material, further inquiries can be directed to the corresponding author/s.

## Ethics Statement

The studies involving human participants were reviewed and approved by Western University Health Sciences Research Ethics Board. The patients/participants or their legal representatives provided their written informed consent to participate in this study.

## Author Contributions

MS, CM, and CE contributed to conception and design of the study. MS collected the data and wrote the first draft. MS and OM analyzed the data. MS, OM, CM, and CE wrote sections of the manuscript. All authors contributed to manuscript revision, read, and approved the submitted version.

## Conflict of Interest

The authors declare that the research was conducted in the absence of any commercial or financial relationships that could be construed as a potential conflict of interest.
