# Supplementary material for: Cerebral Blood Flow Deviations in Critically Ill Patients: Potential Insult Contributing to Ischemic and Hyperemic Injury
Source: Front Med (Lausanne). 2021 Jan 20;7:615318. doi: 10.3389/fmed.2020.615318 (PMC7854569; doi:10.3389/fmed.2020.615318)
Supplement: Supplementary file 1 [file Table_1.DOCX]

**SUPPLEMENTARY MATERIAL**

1. **Calculating the proportion of time with CBFv deviations above/below thresholds**

Figure SM-1 below demonstrates the method used to calculate the proportion of time that CBFv deviated from baseline above or below thresholds of 5, 10, 15, 20, 25 and 30%.

**Figure SM-1: Method for computing the proportion of time that patient’s CBFv (black line) deviated from baseline above and below pre-determined thresholds (blue shaded areas).**

The three panels above are taken from a single 20-min window from one patient and show CBFv data plotted versus time. The total time that CBFv deviates from baseline into shaded areas in each 20-min window is shown in blue (both absolute and percent of total).

1. **Calculating index of dynamic cerebral autoregulation Mxa**

Figure SM-2 below describes the method to compute index of dynamic autoregulation (Mxa)^28^ from CBFv and MAP data. We used a moving time window of various length that was advanced in 1-minute steps to calculate Spearman’s correlation coefficient between CBFv and MAP values. Disturbed autoregulation was defined as Mxa values >0.3 that were statistically significant (p<0.05).^29^ To determine the impact of window length on calculation of Mxa, we varied the window length across a range of values (5, 10, 15, 20, 30 and 60 minutes). Mxa was only computed in windows that had at least 5 mmHg variation in MAP.^30^

Figure SM-2: Sample data from a representative patient showing computation of Mxa.

Panel A shows 20-minute tracing of CBFv and MAP from a representative patient. Two 5-minute windows are shown to demonstrate periods where autoregulation was impaired (red) versus preserved (green). Panel B zooms in on CBFv and MAP data from the red window in Panel A. Panel C shows Spearman correlation of data from Panel B, with Mxa (r=0.738) exceeding threshold (>0.3) and correlation being statistically significant (p < 0.001). This suggest impaired dynamic cerebral autoregulation for the selected window. Panel D zoom zooms in on CBFv and MAP data from the green window in Panel A. Panel E shows Spearman correlation of data from Panel D, with Mxa (r=0.038) below threshold (>0.3) and non-significant correlation (p < 0.772), suggesting preserved dynamic cerebral autoregulation for the selected window.

1. ***Regression analysis to determine the relative impact of MAP and CO_2_ on CBFv***

Figure SM-3: Sample regression analysis from a representative patient. Panel A shows CBFv, MAP and PetCO_2_ data for the entire duration of the recording. Panel B shows a 5-min moving window (same as used to calculate Mxa) from which multivariable regression is calculated (Panel C) revealing low R^2^ for this window. Panel D shows an adjacent 5-min window (2 minutes across), where again multivariable regression is calculated (Panel E), revealing slightly higher, yet still small, R^2^. This analysis was repeated for each patient using moving windows of various lengths.

Table SM-1: Within patient results of the multivariable linear regression analysis with MAP and CO2 and independent variables and CBFv as a dependent variable.

|  | **Window length (min)** | | | | | |
| --- | --- | --- | --- | --- | --- | --- |
| **Patient** | **5** | **10** | **15** | **20** | **30** | **60** |
| 1 | 0.166 ± 0.129 | 0.166 ± 0.129 | 0.164 ± 0.160 | 0.18 ± 0.177 | 0.205 ± 0.197 | 0.234 ± 0.216 |
| 2 | 0.176 ± 0.179 | 0.192 ± 0.185 | 0.210 ± 0.184 | 0.219 ± 0.184 | 0.217 ± 0.184 | 0.173 ± 0.170 |
| 3 | 0.323 ± 0.276 | 0.390 ± 0.279 | 0.419 ± 0.275 | 0.451 ± 0.274 | 0.506 ± 0.269 | 0.583 ± 0.237 |
| 4 | 0.125 ± 0.119 | 0.106 ± 0.118 | 0.100 ± 0.104 | 0.106 ± 0.109 | 0.115 ± 0.124 | 0.138 ± 0.157 |
| 5 | 0.081 ± 0.085 | 0.051 ± 0.054 | 0.043 ± 0.040 | 0.039 ± 0.032 | 0.035 ± 0.029 | 0.028 ± 0.032 |
| 6 | 0.148 ± 0.162 | 0.107 ± 0.125 | 0.102 ± 0.134 | 0.097 ± 0.140 | 0.092 ± 0.127 | 0.079 ± 0.091 |
| 7 | 0.108 ± 0.141 | 0.094 ± 0.104 | 0.110 ± 0.128 | 0.127 ± 0.142 | 0.160 ± 0.164 | 0.197 ± 0.162 |
| 8 | 0.060 ± 0.073 | 0.043 ± 0.062 | 0.038 ± 0.052 | 0.035 ± 0.048 | 0.032 ± 0.042 | 0.027 ± 0.022 |
| 9 | 0.181 ± 0.180 | 0.184 ± 0.207 | 0.202 ± 0.228 | 0.224 ± 0.246 | 0.269 ± 0.269 | 0.333 ± 0.272 |
| 10 | 0.198 ± 0.178 | 0.186 ± 0.172 | 0.186 ± 0.176 | 0.184 ± 0.183 | 0.195 ± 0.184 | 0.213 ± 0.188 |
| 11 | 0.119 ± 0.146 | 0.157 ± 0.185 | 0.196 ± 0.209 | 0.213 ± 0.214 | 0.229 ± 0.214 | 0.279 ± 0.189 |
| 12 | 0.100 ± 0.106 | 0.066 ± 0.081 | 0.050 ± 0.062 | 0.043 ± 0.055 | 0.039 ± 0.048 | 0.042 ± 0.045 |
|  |  |  |  |  |  |  |
| **Group mean ± SD** | **0.133 ± 0.153** | **0.141 ± 0.178** | **0.154 ± 0.192** | **0.165 ± 0.204** | **0.182 ± 0.219** | **0.205 ± 0.231** |

1. ***Duration of CBFv deviations above/below thresholds***

Table SM-2 summarizes the proportion of analysis time that CBFv was above or below prespecified thresholds across patients. Data are shown as mean ± standard deviations.

Table SM-2: Proportion of analysis time CBFv was above or below specific thresholds

|  | Proportion of analysis time that CBFv was | | |
| --- | --- | --- | --- |
| CBFv Threshold  (% from baseline) | Above threshold | Below threshold | Above & Below threshold |
| 5 | 0.43 ± 0.31 | 0.31 ± 0.23 | 0.74 ± 0.15 |
| 10 | 0.31 ± 0.31 | 0.21 ± 0.2 | 0.52 ± 0.24 |
| 15 | 0.2 ± 0.31 | 0.14 ± 0.16 | 0.34 ± 0.28 |
| 20 | 0.15 ± 0.31 | 0.09 ± 0.13 | 0.24 ± 0.3 |
| 25 | 0.13 ± 0.31 | 0.06 ± 0.11 | 0.19 ± 0.3 |
| 30 | 0.12 ± 0.3 | 0.05 ± 0.1 | 0.17 ± 0.29 |

1. ***Duration of disturbed dynamic autoregulation***

Table SM-3 summarizes the duration of disturbed autoregulation across patients expressed as the fraction of the total analysis time. The results are shown for different moving window lengths used to compute Mxa. Data are shown as mean ± standard deviations.

Table SM-3: Fraction of analysis time with disturbed autoregulation across patients

| **Window Length**  **(min)** | **Fraction of analysis time with**  **disturbed autoregulation**  **(as % of total observation time)** |
| --- | --- |
| 5 | 20± 16% |
| 10 | 23 ± 20% |
| 15 | 25 ± 22% |
| 20 | 27± 24% |
| 25 | 30 ± 25% |
| 30 | 35 ± 28% |

1. ***Relationship between CBFv deviations and state of dynamic autoregulation***

Figures SM-4 and SM5 show cumulative time (expressed as a fraction of the 5-min windows used to compute Mxa) that CBFv deviated above and below thresholds when autoregulation was impaired versus preserved for each individual patient.

Figure SM-4: Relationship between CBFv deviations above/below thresholds and state of dynamic autoregulation (patients 1-6)

Figure SM-5: Relationship between CBFv deviations above/below thresholds and state of dynamic autoregulation (patients 7-12)

Table SM-4: Relationship between CBFv deviations above/below thresholds and state of dynamic autoregulation across patients.

|  | **Time CBFv ABOVE threshold**  **(fraction of time in the 5-min window used to compute Mxa)** | | | **Time CBFv BELOW threshold**  **(fraction of time in the 5-min window used to compute Mxa)** | | |
| --- | --- | --- | --- | --- | --- | --- |
|  | **AR Preserved** | **AR Impaired** |  | **AR Preserved** | **AR Impaired** |  |
| **CBF threshold** | **mean ±**  **SD** | **mean ±**  **SD** | **p-value** | **mean ±**  **SD** | **mean ±**  **SD** | **p-value** |
| 5 | 0.449 ± 0.306 | 0.398 ± 0.217 | 0.9979 | 0.309 ± 0.230 | 0.342 ± 0.213 | 0.9975 |
| 10 | 0.335 ± 0.324 | 0.268 ± 0.194 | 0.9899 | 0.218 ± 0.206 | 0.246 ± 0.189 | 0.9989 |
| 15 | 0.212 ± 0.314 | 0.181 ± 0.204 | 0.9999 | 0.152 ± 0.178 | 0.172 ± 0.163 | 0.9999 |
| 20 | 0.152 ± 0.318 | 0.125 ± 0.214 | >0.9999 | 0.104 ± 0.149 | 0.108 ± 0.141 | >0.9999 |
| 25 | 0.138 ± 0.313 | 0.100 ± 0.210 | 0.9996 | 0.07 ± 0.126 | 0.071 ± 0.127 | >0.9999 |
| 30 | 0.128 ± 0.305 | 0.086 ± 0.202 | 0.9993 | 0.052 ± 0.115 | 0.056 ± 0.117 | >0.9999 |
